# Evaluating the spatial accessibility and spatial layout optimization of HIV/AIDS healthcare services in Shandong Province, China

**DOI:** 10.1038/s41598-024-61484-7

**Published:** 2024-05-17

**Authors:** Chao Zhang, Yujie Yan, Xiaoyan Zhu, Ling Li, Yajun Li, Guoyong Wang, Fenfen He, Yining Song, Yunxia Liu, Na Zhang

**Affiliations:** 1 Department of Biostatistics, School of Public Health, Cheeloo College of Medicine, Shandong University, Jinan, 250012 Shandong China; 2 Institute for Medical Dataology, Cheeloo College of Medicine, Shandong University, Jinan, 250000 Shandong China; 3 Shandong Center for Disease Control and Prevention, Jinan, 250014 Shandong China; 4 Department of Occupational and Environmental Health and the Ministry of Education Key Lab of Hazard Assessment and Control in Special Operational Environment, School of Public Health, Fourth Military Medical University, Xi’an, China; 5 Climate Change and Health Center, Shandong University, Jinan, Shandong Province China

**Keywords:** Spatial accessibility, Multi-modal 2SFCA, HIV/AIDS healthcare service, Spatial layout optimization, Health policy, Health services, HIV infections

## Abstract

Improving access to HIV/AIDS healthcare services is of great concern to government and policymakers striving to strengthen overall public health. How to reasonably allocate HIV/AIDS healthcare resources and maximize the equality of access to healthcare services across subdistrict areas has become an urgent problem to be solved. However, there is limited research on this topic in China. It is necessary to evaluate spatial accessibility to improve the accessibility and equity of HIV/AIDS healthcare services. In this study, the improved multi-modal two-step floating catchment area (2SFCA) and inverted 2SFCA (i2SFCA) methods are used to measure the spatial accessibility of HIV/AIDS healthcare services and the crowdedness of the healthcare sites in Shandong Province, China. Then, the theoretical supply and the optimal spatial distribution of resources are calculated and visualized by minimizing the accessibility gaps between demand locations. This study showed that the spatial accessibility of HIV/AIDS service resources in Shandong Province was concentrated and unevenly distributed, and the accessibility scores in the marginal areas of prefecture-level cities were significantly lower than those in other areas. Regions with a large number of doctors had significantly higher levels of spatial accessibility. The ART accessibility scores in the southwest of Shandong Province were higher than those in other regions. As the travel friction coefficient increased, the accessibility scores formed an approximately circular cluster distribution centered on the healthcare sites in geographical distribution. More ART drugs needed to be supplied in marginal areas and more doctors were needed to work on HIV/AIDS in urban areas to address the spatial distribution imbalance of HIV/AIDS healthcare services. This study profoundly analyzed the spatial accessibility of HIV/AIDS healthcare services and provided essential references for decision-makers. In addition, it gives a significant exploration for achieving the goal of equal access to HIV/AIDS healthcare services in the future.

## Introduction

Acquired immune deficiency syndrome (AIDS) is an immunodeficiency disease caused by human immunodeficiency virus (HIV) infection^1^. There were a total of 85.6 million people living with HIV (PLWH), and 40.4 million people have died from AIDS-related illnesses since the start of the epidemic^2^. In China, by the end of 2020, there were more than1 million PLWH, with a cumulative reported deaths of 351,000^3^. Despite Shandong Province having a lower HIV/AIDS incidence rate than the national average, it is the second most populous province in China with developed transportation. Its large population and strong mobility are easy to cause the spread of the HIV^4,5^. By the end of 2022, there are still 2,9451 PLWH in Shandong Province.

Until now, there is no effective vaccine to prevent AIDS and no effective treatment to cure AIDS. The PLWH need long-term antiretroviral therapy (ART) to restore their immune system. ART can improve the quality of life and extend the life expectancy of PLWH by inhibiting the virus^6,7^. Therefore, the daily treatment and care of PLWH is a complex and long-term work. With the increase in the number of PLWH, the demand for ART drugs is also increasing, and the workload of doctors in healthcare sites is also gradually increasing. Previous studies have shown that the insufficient number and overburden of doctors in HIV/AIDS healthcare sites will be detrimental to the provision of effective ART and reduce the quality of care^8,9^. High quality and continuous care are conducive to PLWH receiving ART and improving their adherence to ART^10^.In addition, there is also inequalities in accessibility and uptake of HIV services^11,12^. These disparities often stem from various socio-economic, geographical, and institutional barriers that prevent certain groups within the population from obtaining the necessary healthcare. For instance, marginalized communities, including low-income families and those living in remote or rural areas, frequently face difficulties in accessing HIV testing, treatment, and counseling services^13^. Consequently, these inequalities not only hinder the effective management and control of AIDS but also contribute to the continued spread of the virus, underscoring the urgent need for targeted interventions to ensure equitable access to HIV/AIDS healthcare services for all.

The spatial accessibility is closely related to people's demand for public service facilities, and can comprehensively consider the spatial layout, transportation network, geographical distance and other factors between public service facilities and residents. The spatial accessibility of healthcare service facilities, particularly those related to HIV/AIDS healthcare services, has received considerable attention in recent years^14,15^. Previous studies showed that long distances, unreasonable resource allocation, and spatial layout will make it more difficult to obtain HIV/AIDS healthcare services^16,17^. However, there is limited research on this topic in China. In addition, the rapid urbanization seen in many parts of China potentially wide the accessibility gap between densely populated cities and more remote areas, it becomes imperative to delve deeper into the spatial accessibility of HIV/AIDS healthcare services in China.

Understanding the crucial role geographic location plays in accessing HIV/AIDS healthcare services is essential for identifying underserved regions and populations^18,19^. Research from the United States highlights spatial accessibility as a significant barrier in HIV/AIDS management among PLWH^20^. In this regard, the two-step floating catchment area (2SFCA) method stands out as an effective analytical tool, offering a detailed view on accessibility by considering the scale of supply, demand, and the distance between supply and demand^21–26^. Its extensive application in assessing healthcare accessibility^27,28^ underscores its value, particularly for diseases like HIV/AIDS that may have specific service requirements. Moreover, the 2SFCA method has been subject to continuous refinements, including multi-modal 2SFCA proposed by various scholars^29^. The multi-modal 2SFCA acknowledges the diverse transportation options available to individuals, offering a more comprehensive view of spatial accessibility. This improvement highlights the method's adaptability to complex, real-world scenarios.

This study aims to measure the spatial accessibility of HIV/AIDS healthcare services in Shandong Province in 2022 by using the improved multi-modal 2SFCA method. Then, by minimizing the accessibility gaps between demand locations, the optimal spatial distribution of theoretical supply and resources was calculated and visualized. It is expected to identify areas with insufficient spatial accessibility and to make policy recommendations for strengthening HIV/AIDS healthcare services.

## Data and methods

### Study area

**Figure 1:**
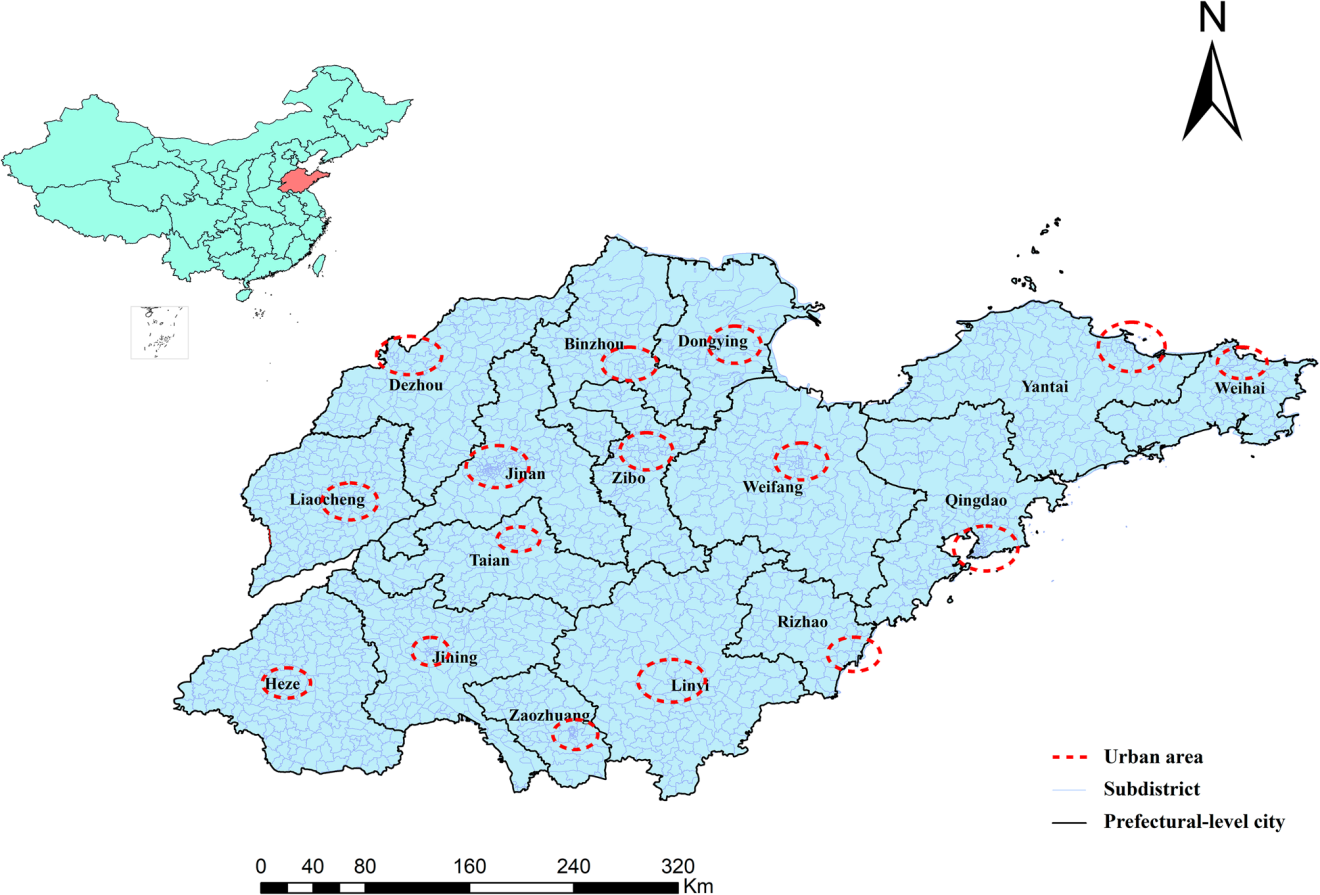
The location and administrative divisions of Shandong Province. (Sources: http://www.geodata.cn/, the map was edited using ArcGIS 10.8).

Shandong Province, with a total area of 158,000 km^2^, is located in eastern China, 34°25′to 38°23′ N latitude and 114°36′ to 122°43′ E longitude. With approximately 100 million residents, it is the second-largest province in China. Shandong Province is a relatively developed region in China with a gross domestic product of about 8.31 billion in 2021. Figure 1 shows the 16 prefectural-level cities and 1984 subdistricts in 2021. Jinan and Qingdao are the most developed and densely populated cities in Shandong Province. The relative geographic information was collected from the National Center for Earth System Science and Data (http://www.geodata.cn/).

### Data collection

Data on PLWH were extracted from Shandong Provincial HIV/AIDS Comprehensive Response Information Management System (CRIMS)^30,31^. CRIMS is a powerful tool for HIV/AIDS testing, treatment, and management in China^32^, and contains the demographic characteristics of PLWH, including date of birth, gender, time of death, geocoding of residence, etc.

The list and address of HIV/AIDS healthcare sites in Shandong Province were obtained from the official website of the Health Commission of Shandong Province (http://wsjkw.shandong.gov.cn/). The coordinates of these sites were collected based on their addresses using the Baidu Map Geocoding Application Programming Interface (API). The number of doctors and the consumption of ART drugs in 2022 were also collected for subsequent analysis to describe the supply capacity and the crowdedness of HIV/AIDS healthcare sites. The population data used in this study were obtained from the LandScan population spatial raster dataset (https://landscan.ornl.gov) with a resolution of 1 km in 2022. The dataset was developed by the Oak Ridge National Laboratory and provided by East View Cartographic. Due to the uneven spatial distribution of the population, the population-weighted subdistrict centroids were used to denote the demand locations.

### Methods

#### The generalized 2SFCA framework

The 2SFCA method is used to assess spatial accessibility, which involves two steps^33^. In the first step, the algorithm searches for all demand locations within the catchment area of each HIV/AIDS healthcare site in this study. Then, the supply–demand ratio for each site is calculated, i.e., the average supply per PLWH. In the second step, the supply–demand ratios of all healthcare sites within the catchment region of each demand location are aggregated. The spatial accessibility score of the demand location is determined by the sum of the supply–demand ratios of each demand location.

The original 2SFCA method adopts a dichotomous distance decay function^33^, which has a major limitation^34^. Several additional distance decay functions, such as the Gaussian function^35^, the power function^36^, and the Kernel density function^37^, have been developed to address this limitation. These improvements can be unified by a generalized 2SFCA framework developed by Wang^26^:

$${A}_{i}={\sum }_{j=1}^{n}\frac{{S}_{j}f\left({d}_{ij}\right)}{{\sum }_{k=1}^{m}\left({D}_{k}f\left({d}_{kj}\right)\right)}$$

where ${A}_{i}$ is the spatial accessibility score at demand location $i$, ${S}_{j}$ is the supply capacity at location $j$, ${D}_{k}$ is the demand amount, ${d}_{ij}\left({d}_{kj}\right)$ is the distance or travel time between $i$ ($k$) and $j$, $n$ is the total number of subdistricts, $f$ is a general distance decay function. In this study, the gravity model with a power function, in which no distance threshold was set, was adopted as the distance decay function^25^, since the necessity of ART for PLWH. The power function can be formulated as:

$$f\left({d}_{ij(il)}\right)= {d}_{ij(il)}^{-\beta }$$

where $\beta$ is the travel friction coefficient, which represents the resistance of people to arrive at the healthcare sites. We chose multiple values within the range [0.5, 2.0] for the *β* to explore how changes in these travel friction factors influence the accessibility of HIV/AIDS healthcare services.

The number of doctors involved in AIDS-related work (e.g., doctors per 1000 PLWH) and the supply of ART drugs (e.g., the boxes of ART drugs per PLWH) were set as indicators to evaluate the score of accessibility to HIV/AIDS services in a subdistrict.

#### The inverted 2SFCA framework

As mentioned above, the 2SFCA method calculates the supply–demand ratio for each healthcare site. If the demand–supply ratios are calculated, an evaluation of the supply demands of the healthcare sites can be obtained, which can be intuitively understood as the "potential crowdedness" of the healthcare sites^38^. This calculation method is called the inverted 2SFCA (i2SFCA) method:

$${C}_{j}={\sum }_{i=1}^{m}\frac{{D}_{i}f\left({d}_{ij}\right)}{\sum_{l=1}^{n}\left({S}_{l}f\left({d}_{il}\right)\right)}$$

where ${C}_{j}$ is the potential crowdedness at HIV/AIDS healthcare site $j$, ${S}_{l}$ is the supply capacity at location $l$, ${D}_{i}$ is the demand amount, ${d}_{ij}\left({d}_{il}\right)$ is the travel time between $i$ and $j (l)$, $m$ is the total number of healthcare sites, $f$ is the power distance decay function. In this study, the potential crowdedness of a healthcare site is measured by two indicators, the ART drugs supply demands (e.g., PLWH per 1,000 boxes of ART drugs) and the workload of its doctors (e.g., PLWH per doctor).

#### The multi-modal 2SFCA method

Most applications of the 2SFCA method currently in use typically account for only one mode of transportation, often private automobile^39^. Such studies overlook the reality that people may access services (like healthcare facilities) through a variety of transport options including private cars, taxis, subways, or buses. In this study, we used the multi-modal 2SFCA method as improved by Tao et al.^29^, which is based on the generalized 2SFCA. The comprehensive explanation of the method's formulation and the detailed procedural steps are described in the supplementary material Text S1.

#### Estimating the distance of each subdistrict to HIV/AIDS healthcare sites

In this study, we utilized the APIs provided by Baidu Map (lbsyun.baidu.com/index.php) through Python programming to get a reliable evaluation of drive time and public transit time^40,41^. Meanwhile, we have considered China's regional jurisdiction policy on infectious disease control, which encourages PLWH to seek HIV/AIDS healthcare services within the prefecture-level of their respective cities. The travel times required for PLWH to reach a healthcare site in their city of residence were calculated.

#### Measuring accessibility and formulating the planning

Use $S\left({S}_{1}+{S}_{2}+\dots +{S}_{n}\right)$ to represent the total supply and $D\left({D}_{1}+{D}_{2}+\dots +{D}_{m}\right)$ to represent the total demand. As the following equation shows, the accessibility score ($\alpha$) is that its weighted mean is equal to the ratio of total supply to total demand in the study area^42^.

$$\alpha =\sum_{i=1}^{m}(\frac{{D}_{i}}{D}){A}_{i}=S/D$$

The deviation of actual accessibility from $\alpha$ is used to measure inequality. We utilized the least squares method to minimize the variance of the accessibility index ${A}_{i}$ across all demand locations $i$ by redistributing the supply ${S}_{j}$ among the supply locations $j$^37^.

$$min={\sum_{i=1}^{m}{D}_{i}({A}_{i}-\alpha )}^{2}$$

In this study, all figures in this study were produced with ArcGIS 10.8 (Esri, https://www.esri.com/en-us/arcgis/products/arcgis-desktop/overview). The figure generation process involved data importation, spatial analysis, and cartographic design within the software. Further, spatial Kriging interpolation analysis was conducted in ArcGIS 10.8 to generate a continuous surface of spatial accessibility distribution. An automated ArcGIS toolkit developed by Zhu and Wang^43^ was used to implement the 2SFCA and i2SFCA. Python 3.11 was used to formulate the related planning of spatial layout optimization.

#### Ethics approval and consent to participate

All methods in our study were carried out in accordance with relevant guidelines and regulations. The protocol was approved by the ethics committee at the Shandong Centers for Disease Control and Prevention (CDC) (2021–50). All subjects and/or their legal guardians provided verbal informed consent.

## Results

### The spatial distribution of PLWH and HIV/AIDS healthcare services

**Figure 2:**
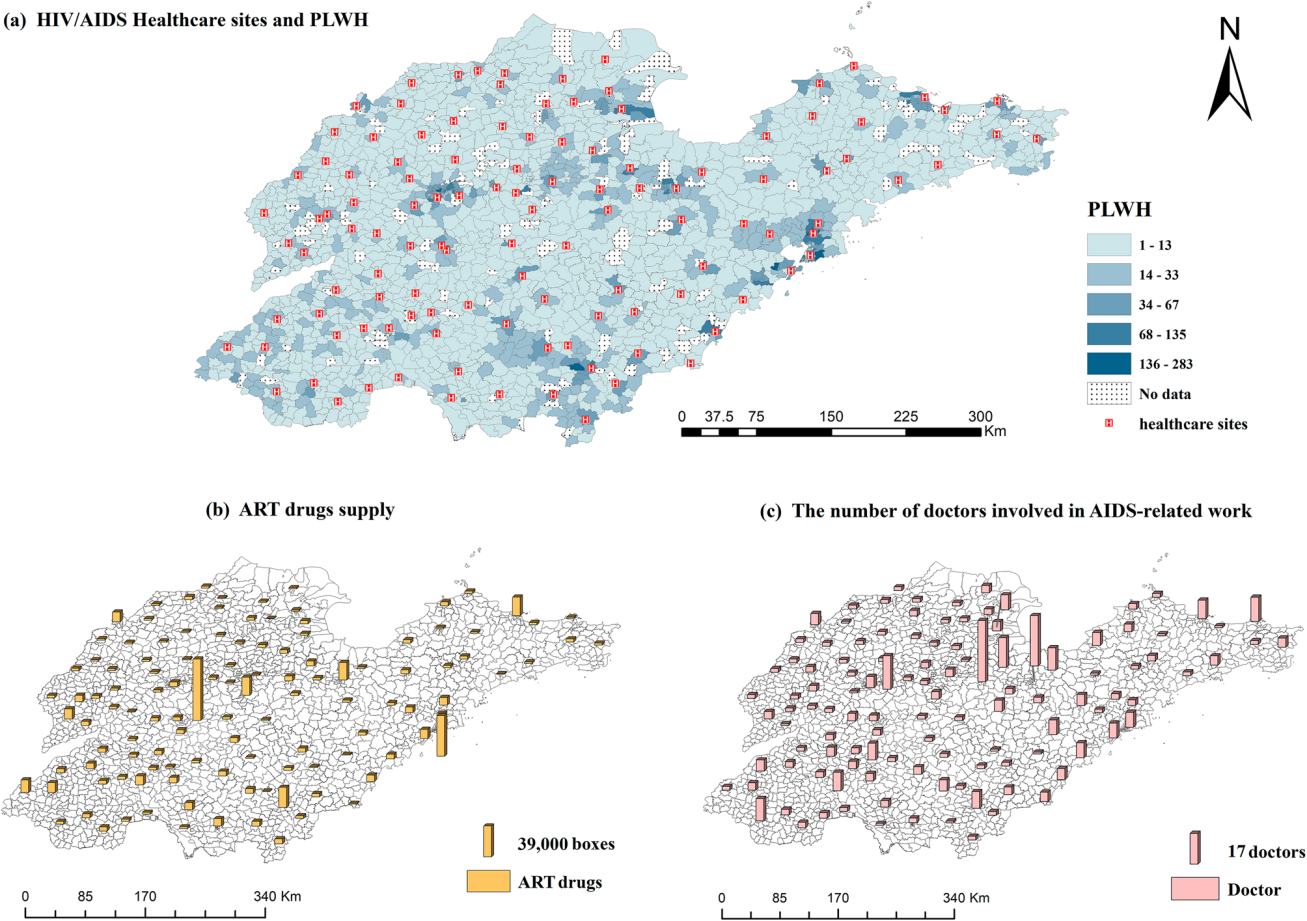
The spatial distribution of PLWH, HIV/AIDS healthcare sites, ART drugs supply, and doctors in Shandong province in 2022. Areas of the map with darker blue color in (**a**) represent higher number of PLWH. The yellow columns in (**b**) represent the number of doctors involved in AIDS-related work, and the pink columns in (**c**) represent the supply of ART drugs. (Sources: http://www.geodata.cn/, the map was edited using ArcGIS 10.8).

Figure 2a displays the spatial distribution of PLWH and HIV/AIDS healthcare sites in Shandong Province. Due to the extensive time range of the data and the alterations in subdistrict divisions, 1676 subdistricts out of 1984 were successfully matched. In 2022, a total of 23,988 PLWH in Shandong Province received HIV/AIDS healthcare services. Qingdao and Jinan accounted for 16.7% and 13.5% of the total, respectively (Table S1). Additionally, as depicted in Fig. 2b, Shandong Province has a network of 118 HIV/AIDS healthcare sites, distributed in different geographical locations. In 2022, a total of 680,204 boxes of ART drugs were consumed at HIV/AIDS healthcare sites. Qingdao and Jinan accounted for 15.6% and 14.2% of the total supply, respectively, ranking the top two. Figure 2c depicts the number of doctors involved in AIDS-related work. The total number of doctors was 492, and Weifang and Jining were in the leading position, accounting for 23.2% and 9.1% of the total, respectively.

### Spatial accessibility to HIV/AIDS healthcare services and crowdedness of healthcare sites

**Figure 3:**
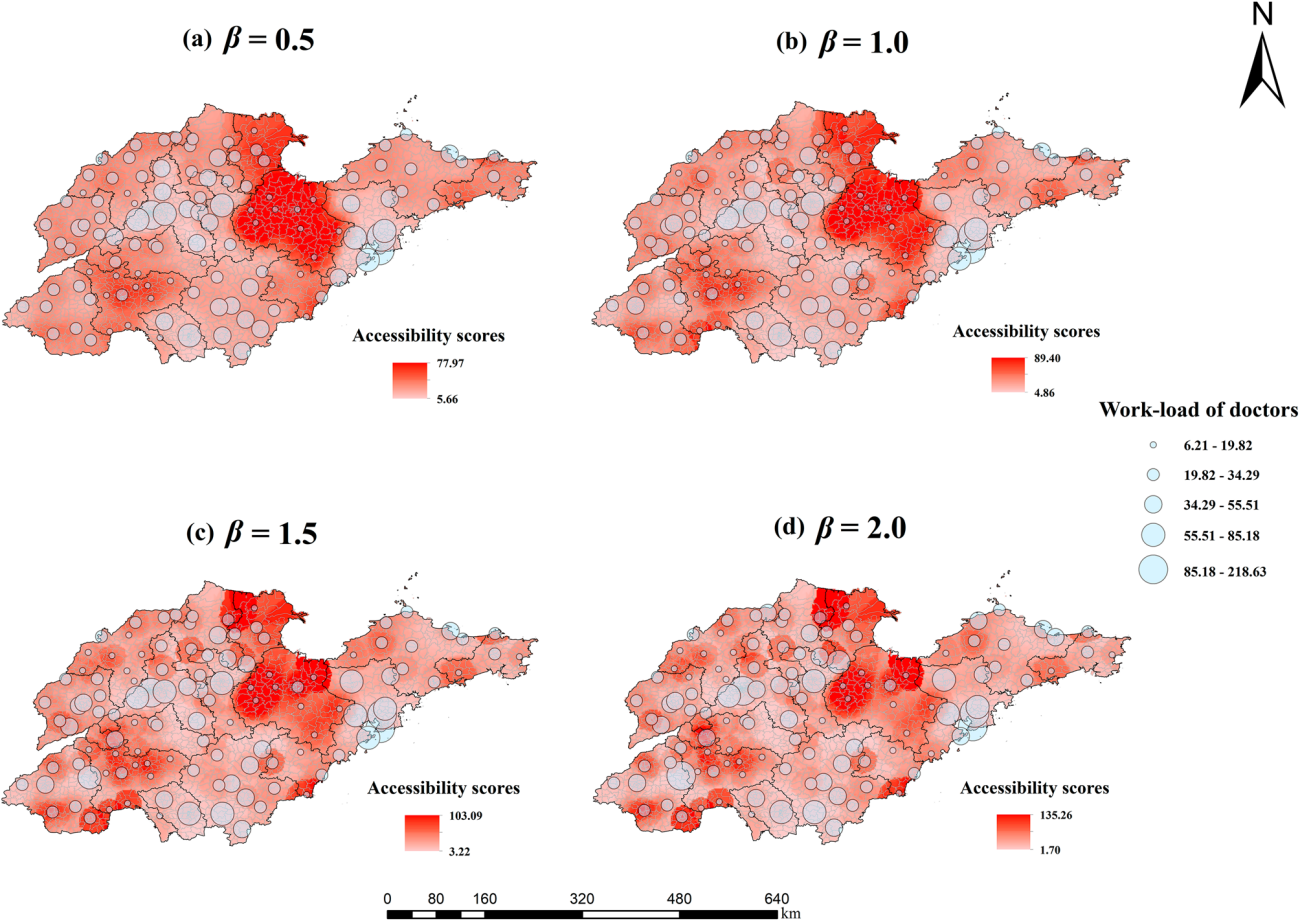
Spatial accessibility scores to doctors and workload of doctors in the HIV/AIDS healthcare sites when (**a**) *β* = 0.5, (**b**) *β* = 1.0, (**c**)* β* = 1.5, and (**d**) *β* = 2.0. The size of the blue circles represents the four levels of work-load of doctors. Areas of the map with darker red color represent higher accessibility scores of doctors. (Sources: http://www.geodata.cn/, the map was edited using ArcGIS 10.8).

**Figure 4:**
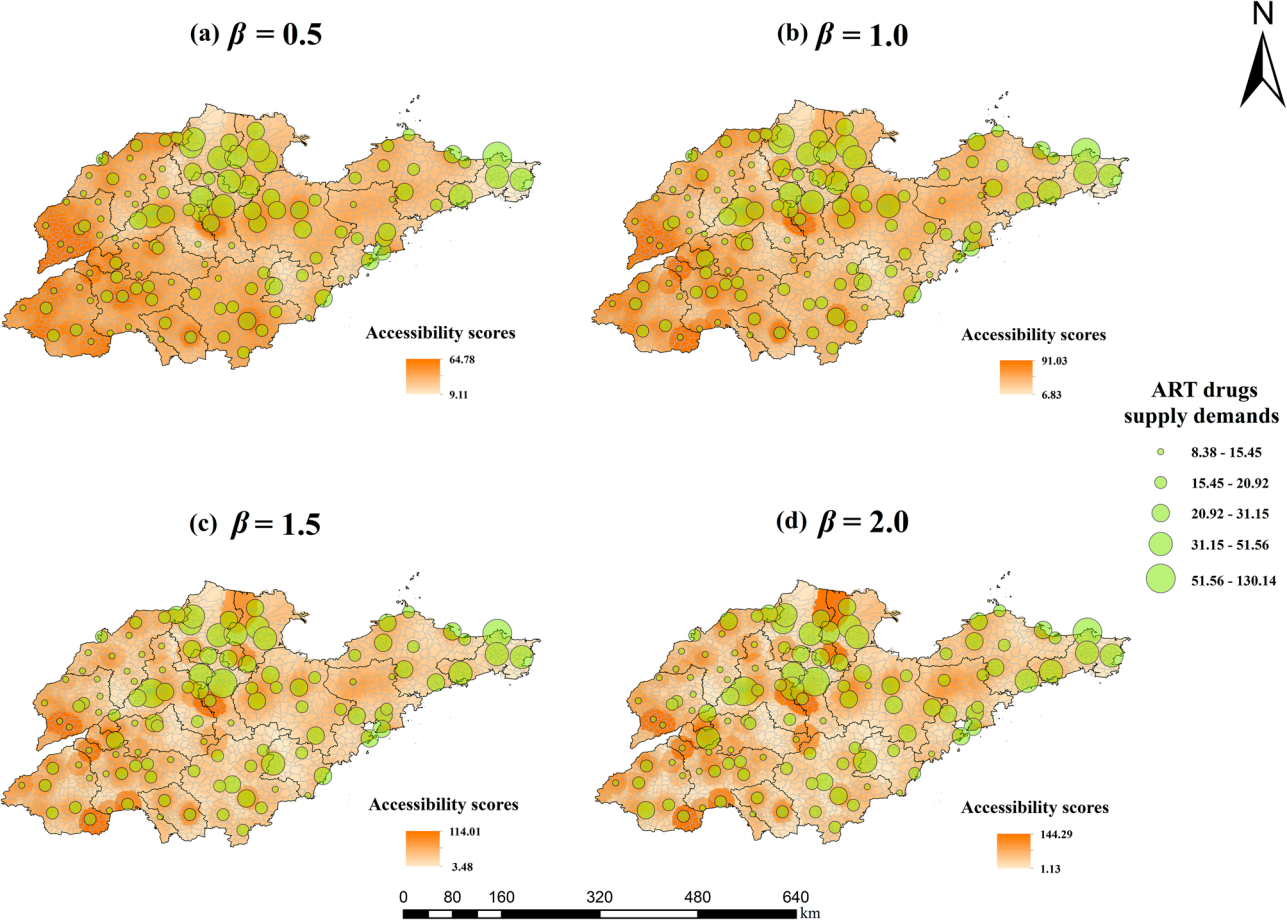
Spatial accessibility scores to ART and ART drugs supply demands in the HIV/AIDS healthcare sites when (**a**) *β* = 0.5; (**b**) *β* = 1.0; (**c**) *β* = 1.5; and (**d**)* β* = 2.0. The size of the green circles represents the four levels of the ART drugs supply demands. Areas of the map with darker orange color represent higher accessibility scores of ART drugs. (Sources: http://www.geodata.cn/, the map was edited using ArcGIS 10.8).

Figure 3 presents the spatial accessibility to doctors and the workload of doctors in HIV/AIDS healthcare sites. It clearly shows that Weifang, Jining, and Dongying, which had a large number of doctors, had much higher accessibility scores. In Jinan and Qingdao, with the largest number of PLWH, the accessibility of almost all subdistricts was typically somewhat poor. In addition, the study shows that the accessibility scores of marginal areas of prefecture-level cities were significantly lower than those of other areas. The urban areas of Jinan, Qingdao, and Zibo are the healthcare sites with the largest workload of doctors in Shandong Province. Figure 4 presents the spatial accessibility to ART and ART drugs supply demands at HIV/AIDS healthcare sites. The accessibility scores of ART drugs in the southwestern region of Shandong were higher than those in other regions, and the cities with high supply demand for ART drugs were mainly distributed in the northern and peninsula regions.

As the travel friction coefficient increased, PLWH living far away from HIV/AIDS healthcare sites were less likely to obtain healthcare services, and their accessibility scores were lower, forming an approximately circular cluster distribution centered around healthcare sites in geographical distribution. With the increase of travel friction coefficient, the levels of crowdedness of healthcare sites changed little in general, only changing at some sites.

### Planning toward equal accessibility to HIV healthcare services in Shandong Province

We chose multiple values within the range [0.5, 2.0] as the travel friction coefficient *β*. The results are summarized in Table 1. The standard deviation of accessibility scores significantly decreases with the decrease of travel friction coefficient, and the range of equalized accessibility scores is much narrower than that of actual accessibility scores. The ratio of the standard deviations between preoptimization and post-optimization for the accessibility scores of doctors and ART was greater than 1.

**Table 1:** The accessibility of actual versus equalized HIV/AIDS healthcare services.

Indicators	Travel friction coefficient	Actual accessibility			Equalized accessibility			Standard deviation ratio
		Min	Max	SD	Min	Max	SD	
Doctors	*β* = 0.5	4.26	112.23	13.43	20.15	25.57	1.11	12.10
	*β* = 1.0	3.43	260.05	18.61	19.35	36.76	3.55	5.24
	*β* = 1.5	2.23	458.68	28.99	19.25	38.22	3.87	7.49
	*β* = 2.0	0.96	754.12	40.65	19.02	41.41	4.57	8.89
ART	*β* = 0.5	7.73	127.18	10.70	26.44	55.19	5.86	1.83
	*β* = 1.0	4.88	322.28	20.26	24.77	78.62	10.98	1.85
	*β* = 1.5	2.07	545.43	34.89	24.22	86.35	12.67	2.75
	*β* = 2.0	0.80	855.72	51.17	24.07	88.48	13.14	3.89

*ART* antiretroviral therapy; *Min* minimum value; *Max* maximum value; *SD* standard deviation.

**Figure 5:**
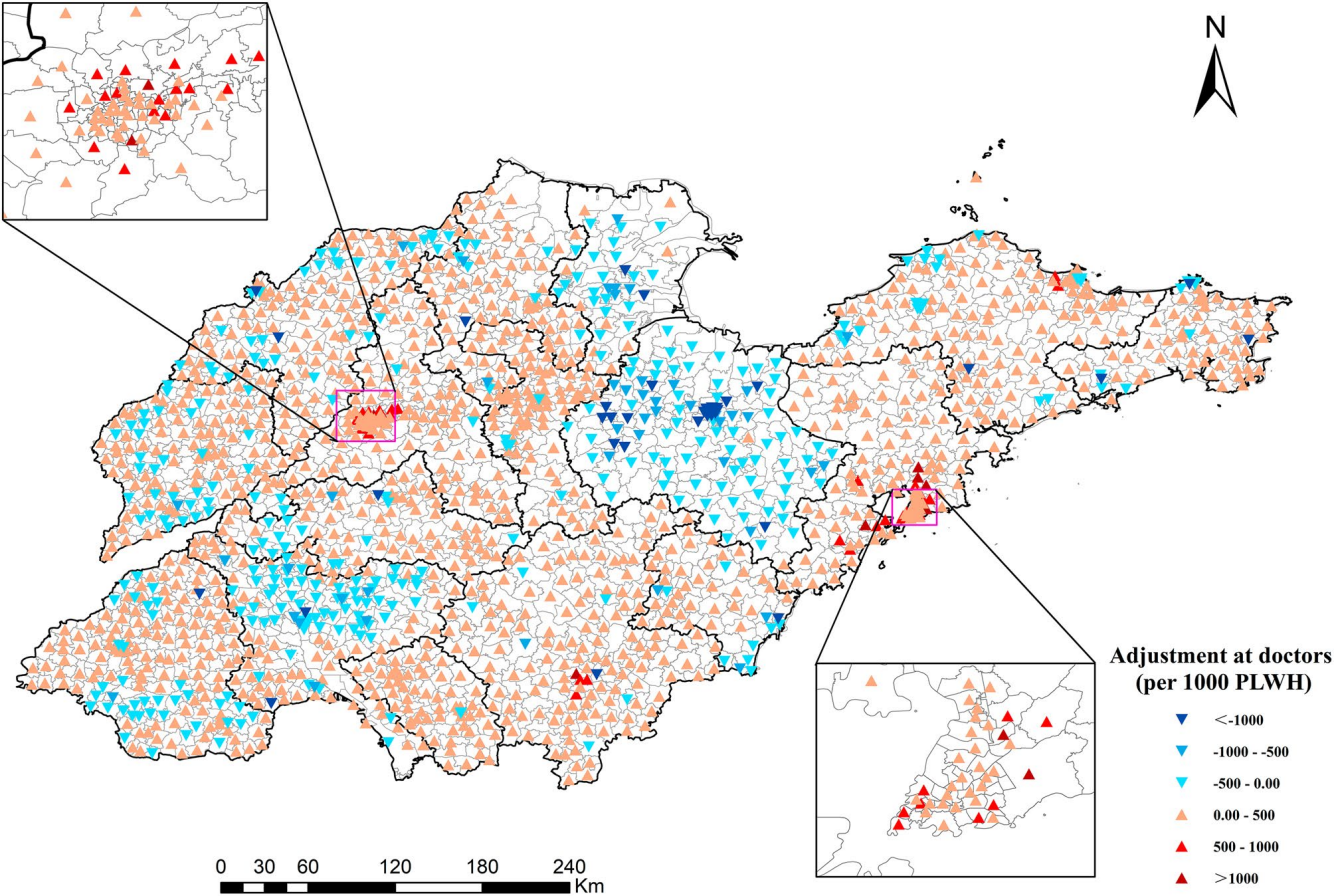
Adjustment of the number of doctors towards equal accessibility (*β* = 1.0). The red points represent the need to increase the number of doctors, while the blue points represent a decrease. (Sources: http://www.geodata.cn/, the map was edited using ArcGIS 10.8).

**Figure 6:**
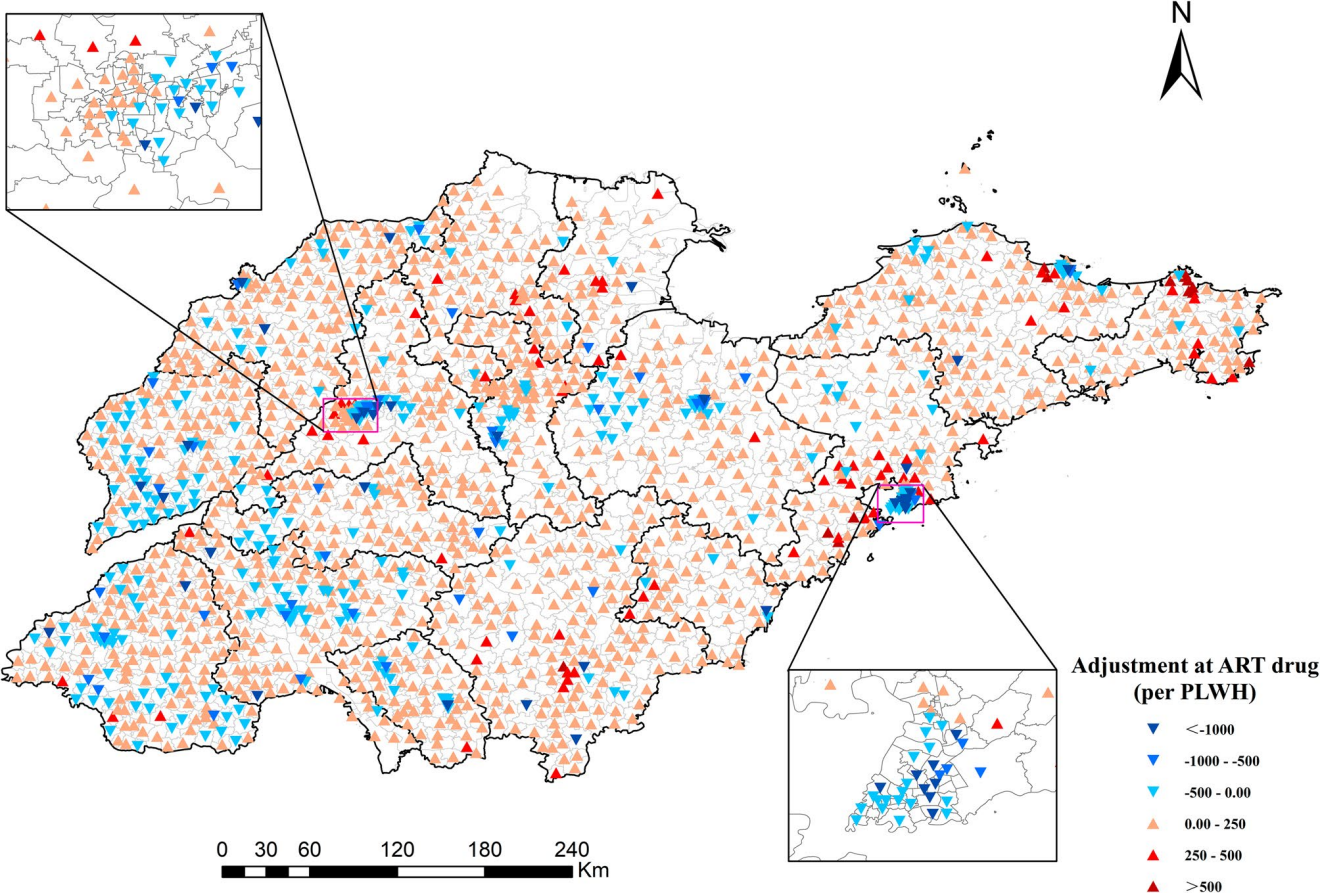
Adjustment at the supply of ART drugs towards equal accessibility (*β* = 1.0). The red points represent the need to increase the supply of ART drugs, while the blue points represent a decrease. (Sources: http://www.geodata.cn/, the map was edited using ArcGIS 10.8).

Taking *β* = 1.0 for example, the differences between the actual and ‘optimized’ doctors and ART are displayed in Figs. 5 and 6, respectively. Most areas of Jining and Weifang are already saturated with doctors, and most subdistricts require 0–500 doctors per 1,000 people, especially in urban areas such as Jinan and Qingdao. In addition, most subdistricts in Shandong province need to increase the supply of ART drugs. Unlike the demand for doctors, the supply of ART drugs in urban areas is relatively saturated, while its supply needs to increase in the marginal areas of prefecture-level cities, such as Zibo, Weihai, Qingdao, etc.

## Discussion

Improving access to HIV/AIDS healthcare services is of great concern to government and policymakers striving to strengthen overall public health. How to reasonably allocate HIV/AIDS healthcare resources and maximize the equality of access to healthcare services across subdistrict areas has become an urgent problem to be solved. In this paper, we used the improved multi-modal 2SFCA method to calculate the accessibility scores of HIV/AIDS healthcare services in the subdistricts of Shandong Province and analyzed the results of the spatial optimal allocation of resources. The 2SFCA with a gravity function is a universal and effective method to assess the accessibility of HIV/AIDS healthcare. It takes into account various spatial factors, assisting in informed decision-making for healthcare planning and resource allocation.

Our study shows that the spatial accessibility of HIV/AIDS service resources in Shandong Province is concentrated and unevenly distributed. The accessibility scores of doctors are strongly impacted by their number, and regions with a large number of doctors have significantly higher levels of spatial accessibility. Meanwhile, this study revealed that compared to other cities, the workloads of doctors in urban areas of cities like Qingdao and Jinan were noticeably heavier. The population density of Shandong Province is one of the main reasons for this imbalance. Compared to remote regions, the urban areas of cities typically have a substantially higher population density. Because of this, there are more PLWH, which increases the workload of doctors. To reduce the workload of doctors, on the one hand, urban areas can establish satellite clinics to provide basic services and, if necessary, refer patients to larger centers. On the other hand, it's essential to invest in establishing new HIV/AIDS healthcare sites or expanding existing sites in underserved regions and encourage doctors to work in these regions.

The southwest region of Shandong Province has a lower level of economic development than other regions, but in general, its ART accessibility scores are higher. This implies that the number of ART drugs and healthcare sites may have a certain spatial distribution pattern. On the contrary, the northern region and the peninsula of Shandong Province exhibit low accessibility scores, coupled with relatively high demands for ART drugs at healthcare sites. These findings emphasize the urgent need for the government to urgently optimize the spatial configuration of healthcare sites and ART drugs supply in these areas. Additionally, due to the impact of regional jurisdiction policies, PLWH living in the marginal areas of prefecture-level cities are encouraged to choose healthcare sites that meet the policy criteria but are far away, which increases the difficulty of obtaining services in marginal areas. Therefore, it is necessary to adapt and find innovative policies to accommodate new HIV/AIDS healthcare models and the changing needs of the population.

To address the imbalance in the spatial distribution of HIV/AIDS care services, more ART drugs need to be supplied in marginal areas and more doctors are needed to work on HIV/AIDS in urban areas. Even in some urban areas, such as the western part of Jinan and the northern part of Qingdao, more doctors need to be added to match the high-density residents there. Meanwhile, establishing mobile clinics to regularly visit underserved areas and offer testing, counseling, and treatment can help bridge the gap in service availability.

People usually think that central cities often have more robust healthcare infrastructure with better-equipped HIV/AIDS healthcare and clinics. When transportation infrastructure is convenient, it suggests that PLWH have more choices and are more willing to travel greater distances to access healthcare services, manifesting as a reduction in the standard deviation of accessibility scores across the geographic space and an increased crowdedness at central cities’ HIV/AIDS service sites. As the friction coefficient, representing traffic resistance, increases, we observe a rise in the standard deviation of accessibility scores among regions. This indicates a preference among people for healthcare sites closer to them as the cost of travel increases, making it more challenging to achieve equitable access to HIV/AIDS healthcare services through redistribution of resources alone. In recent years, the development of network technology has gradually made it possible to implement telemedicine programs that connect people in underserved areas with HIV/AIDS healthcare doctors in larger cities^36^. This can help alleviate the resistance caused by inconvenient transportation and provide quality HIV/AIDS healthcare to people in remote areas.

Previous studies have proposed some measures for the distribution of health personnel to maximize spatial accessibility^44–46^. However, the theory of relocating doctors to remote regions to achieve equal accessibility may be difficult to achieve^37^. Nevertheless, we believe that this study is still feasible, appropriate, and constructive. For instance, our findings may provide approaches such as mobile clinics or rotation schemes that can facilitate access to remote regions without the need for permanent relocation of doctors. Additionally, our findings can be used to predict where relevant doctors should be concentrated in underserved regions to improve access to HIV/AIDS healthcare services.

This study has some limitations. Firstly, our study acknowledges a limitation in determining the most accurate *β* value for assessing traffic resistance due to the lack of comprehensive local transportation and travel pattern data. Future research necessitating detailed field surveys could elucidate a more precise *β* value for Shandong province. Secondly, due to the uniformity in the standard of HIV/AIDS care across various service levels and the strict regulation and limited number of healthcare facilities in China, we have chosen not to use the hierarchical 2SFCA in this study^47^, despite its demonstrated superiority in evaluating spatial accessibility for general healthcare services. Finally, although we can use geospatial information technologies to allocate resources more reasonably, it plays a positive role in achieving the goal of equal access to HIV/AIDS healthcare services. However, solving the imbalance of spatial distribution of HIV/AIDS healthcare services is a long-term endeavor, which is related to the pathological basis of people and their living environment, and is affected by the natural and social environment. At the same time, to satisfy people's demands for HIV/AIDS healthcare convenience, it must also be matched with related policies. Only by addressing all aspects of public health can the effectiveness of AIDS prevention and control be improved.

## Conclusion

This study reveals the unequal distribution of HIV/AIDS healthcare services in Shandong Province, China, with urban areas facing increased doctor workload and an 'overcapacity' of ART drugs supply compared to peripheral regions. By optimizing the spatial layout and taking full account of targeted measures, it was able to ensure that all PLWH enjoy equal access to healthcare services. Our findings will help policymakers improve the delivery of HIV/AIDS healthcare services and contribute to broader discussions on healthcare equity and resource allocation, which will benefit communities and society as a whole.

### Supplementary Information

Supplementary Information.

## Data Availability

All data generated and analysed during the course of this study were available from the corresponding author upon request.
