# Supplementary material for: Evaluating the spatial accessibility and spatial layout optimization of HIV/AIDS healthcare services in Shandong Province, China
Source: Sci Rep. 2024 May 17;14:11258. doi: 10.1038/s41598-024-61484-7 (PMC11099158; doi:10.1038/s41598-024-61484-7)
Supplement: Supplementary file 1 — Supplementary Information. [file 41598_2024_61484_MOESM1_ESM.docx]

**Evaluating the Spatial Accessibility and Spatial Layout Optimization of HIV/AIDS Healthcare Services in Shandong Province, China**

**TextS1**

Tao et al. uniformly formatted the multi-modal 2SCFA method based on the generalized 2SFCA framework. Let $p$ represent the mode of public transportation, and$c$ symbolize the mode of traveling by car; consequently, the multi-modal 2SFCA can be structured as follow:

$$A_{i,p}=\sum_{j=1}^{n} \frac{S_{j}f\left( d_{ij,p} \right)}{\sum_{k=1}^{m} \left( D_{k,p}f\left( d_{kj,p} \right) \right)}$$

$$A_{i,c}=\sum_{j=1}^{n} \frac{S_{j}f\left( d_{ij,c} \right)}{\sum_{k=1}^{m} \left( D_{k,c}f\left( d_{kj,c} \right) \right)}$$

where$S_{j}$ is the supply capacity at location $j$. $A_{i,p}$and $A_{i,c}$ are the transit-mode accessibility of the car-mode accessibility at demand location $i$ respectively. $D_{k,p}$ and $D_{k,c}$ are the public transit-mode population and the car-mode population at demand location $k$ respectively. $d_{ij,p}\left( d_{kj,p} \right)$ and $d_{ij,c}\left( d_{kj,c} \right)$ are the transit-mode and the car-mode travel time from demand location $i(k)$ to facility $j$ respectively.

$f$ is the distance decay function:

$$f\left( d_{ij} \right)= d_{ij}^{-\beta}$$

The combined accessibility at each demand location can be calculated as the weighted average of $A_{i,p}$and $A_{i,c}$ as follows:

$$A_{i}=\frac{D_{i,p}A_{i,p}+D_{i,c}A_{i,c}}{D_{i,p}+D_{i,c}}$$

where $A_{i}$is the combined accessibility at demand location $i$ .

**Tables**

Table S1 provides detailed information on the descriptive results of PLWH, the number of AIDS service stations (including the number of doctors and ART drugs), and the travel time (including drive time and transit time) from the population centroids to the service stations in each subdistrict in 16 prefectural-level cities in Shandong Province in 2022.

Table S1. Basic descriptions of PLWH, HIV/AIDS healthcare services, and mean travel time (standard Deviation) in Shandong province in 2022.

| City | PLWH  *n* (%) | Drive time (minutes)  $M(P_{25}, P_{75})$ | Transit time(minutes)  $M(P_{25}, P_{75})$ | HIV/AIDS healthcare sites  *n* (%) | ART drugs (boxes)  *n* (%) | Doctors  *n* (%) |
| --- | --- | --- | --- | --- | --- | --- |
| Jinan | 3345(13.9%) | 40.69（21.83, 59.00） | 315.73(221.48, 406.87) | 8(6.8%) | 96788(14.2%) | 35(7.1%) |
| Qingdao | 3896(16.2%) | 37.47（22.15, 55.77） | 91.45(57.40, 157.57) | 8(6.8%) | 105810(15.6%) | 35(7.1%) |
| Zibo | 1129(4.7%) | 35.30（20.22, 52.14） | 154.93(111.05, 204.43) | 5(4.2%) | 29445(4.3%) | 14(2.8%) |
| Zaozhuang | 620(2.6%) | 22.03（12.30, 31.92） | 120.82(94.40, 154.39) | 2(1.7%) | 18530(2.7%) | 6(1.2%) |
| Dongying | 606(2.5%) | 27.81（16.45, 38.10） | 123.49(95.23, 160.73) | 6(5.1%) | 14433(2.1%) | 23(4.7%) |
| Yantai | 1859(7.7%) | 52.26（33.17, 67.83） | 65.26(47.95, 65.26) | 9(7.6%) | 47290(7.0%) | 33(6.7%) |
| Weifang | 1869(7.8%) | 41.22（26.00, 58.22） | 64.77(44.22, 110.98) | 9(7.6%) | 53134(7.8%) | 114(23.2%) |
| Jining | 1444(6.0%) | 43.03（24.04, 60.64） | 202.92(141.30, 258.83) | 11(9.3%) | 46221(6.8%) | 45(9.1%) |
| Taian | 853(3.6%) | 37.66（21.83, 52.68） | 117.44(60.31, 175.95) | 6(5.1%) | 26511(3.9%) | 18(3.7%) |
| Weihai | 820(3.4%) | 31.73（18.95, 47.74） | 79.08(55.91, 174.67) | 4(3.4%) | 9784(1.4%) | 25(5.1%) |
| Rizhao | 431(1.8%) | 34.20（22.00, 47.02） | 152.39(102.72, 193.62) | 4(3.4%) | 11164(1.6%) | 14(2.8%) |
| Linyi | 2202(9.2%) | 47.52（31.24, 65.90） | 57.27(38.06, 80.13) | 11(9.3%) | 68050(10.0%) | 35(7.1%) |
| Dezhou | 1142(4.8%) | 44.67（27.98, 63.44） | 92.08(58.06, 194.16) | 11(9.3%) | 34581(5.1%) | 25(5.1%) |
| Liaocheng | 1143(4.8%) | 34.10（22.79, 46.83） | 176.18(66.79, 248.98) | 9(7.6%) | 43950(6.5%) | 21(4.3%) |
| Binzhou | 805(3.4%) | 36.65（24.28, 51.17） | 372.95(265.08, 472.72) | 7(5.9%) | 15052(2.2%) | 16(3.3%) |
| Heze | 1824(7.6%) | 40.46（26.07, 56.85） | 159.85(69.78, 217.76) | 8(6.8%) | 59462(8.7%) | 33(6.7%) |
| Total | 23988 | 40.80（25.22, 58.13） | 121.28(60.35, 225.76) | 118 | 680205 | 492 |

PLWH, people living with HIV; *M*: median; *P_25_*: Lower quartile; *P_75_*: Upper quartile; ART, antiretroviral therapy

Table S2. The ratios of car mode and public transit mode

| City | The number of private cars | Household size | Radio of car mode | Radio of public transit mode |
| --- | --- | --- | --- | --- |
| Jinan | 2852698 | 3315054 | 0.8605 | 0.1395 |
| Qingdao | 3078382 | 3857040 | 0.7981 | 0.2019 |
| Zibo | 1220864 | 1836637 | 0.6647 | 0.3353 |
| Zaozhuang | 790253 | 1317899 | 0.5996 | 0.4004 |
| Dongying | 790797 | 842567 | 0.9386 | 0.0614 |
| Yantai | 1901743 | 2875677 | 0.6613 | 0.3387 |
| Weifang | 2754369 | 3317114 | 0.8304 | 0.1696 |
| Jining | 1743547 | 2877818 | 0.6059 | 0.3941 |
| Taian | 984077 | 1975978 | 0.4980 | 0.5020 |
| Weihai | 895184 | 1232663 | 0.7262 | 0.2738 |
| Rizhao | 783693 | 1145127 | 0.6844 | 0.3156 |
| Linyi | 2935351 | 3991481 | 0.7354 | 0.2646 |
| Dezhou | 1164359 | 1991246 | 0.5847 | 0.4153 |
| Liaocheng | 1253799 | 2108102 | 0.5948 | 0.4052 |
| Binzhou | 1054298 | 1408119 | 0.7487 | 0.2513 |
| Heze | 1593581 | 2952941 | 0.5397 | 0.4603 |
| Total | 25796995 | 37045463 | 0.6964 | 0.3036 |
